# Long-Term Effects of Ionizing Radiation on Gene Expression in a Zebrafish Model

**DOI:** 10.1371/journal.pone.0069445

**Published:** 2013-07-30

**Authors:** Lahcen Jaafar, Robert H. Podolsky, William S. Dynan

**Affiliations:** 1 Institute of Molecular Medicine and Genetics, Georgia Regents University, Augusta, Georgia, United States of America; 2 Center for Biotechnology and Genomic Medicine, Georgia Regents University, Augusta, Georgia, United States of America; 3 Departments of Radiation Oncology and Winship Cancer Institute, Emory University School of Medicine, Atlanta, Georgia, United States of America; 4 Department of Biochemistry, Emory University School of Medicine, Atlanta, Georgia, United States of America; Università degli Studi di Milano, Italy

## Abstract

Understanding how initial radiation injury translates into long-term effects is an important problem in radiation biology. Here, we define a set of changes in the transcription profile that are associated with the long-term response to radiation exposure. The study was performed *in vivo* using zebrafish, an established radiobiological model organism. To study the long-term response, 24 hour post-fertilization embryos were exposed to 0.1 Gy (low dose) or 1.0 Gy (moderate dose) of whole-body gamma radiation and allowed to develop for 16 weeks. Liver mRNA profiles were then analyzed using the Affymetrix microarray platform, with validation by quantitative PCR. As a basis for comparison, 16-week old adults were exposed at the same doses and analyzed after 4 hours. Statistical analysis was performed in a way to minimize the effects of multiple comparisons. The responses to these two treatment regimes differed greatly: 360 probe sets were associated primarily with the long-term response, whereas a different 2062 probe sets were associated primarily with the response when adults of the same age were irradiated 4 hours before exposure. Surprisingly, a ten-fold difference in radiation dose (0.1 versus 1.0 Gy) had little effect. Analysis at the gene and pathway level indicated that the long-term response includes the induction of cytokine and inflammatory regulators and transcription and growth factors. The acute response includes the induction of p53 target genes and modulation of the hypoxia-induced transcription factor-C/EBP axis. Results help define genes and pathways affected in the long-term, low and moderate dose radiation response and differentiate them from those affected in an acute response in the same tissue.

## Introduction

Embryos of the zebrafish (*Danio rerio*) are small, transparent, and available in copious numbers. Zebrafish have wide applications as a vertebrate model organism in radiation biology and other fields [1]. The zebrafish genome encodes homologues of mammalian genes involved in the DNA damage response, inflammation, and other disease-relevant processes. Prior work includes studies of radiation toxicity, adaptive and bystander responses, and radiation modifiers [2]–[8].

Here, we use the zebrafish embryo model to investigate the long-term response to radiation. We exposed embryos to γ-rays at a low dose of 0.1 Gy, corresponding to about 1% of the acute LD_50_ in zebrafish and near the lower limit for measurement of acute cell death *in vivo* [5]. Another cohort was exposed to a moderate dose of 1.0 Gy, which remains far below the level associated with developmental delay or defects [3]. We allowed the embryos to develop for 16 weeks before analysis. As a basis for comparison, we irradiated adults at the same doses, four hours before analysis. We used mRNA expression profiling as a sensitive measure of biological response. Application of this technology in other biological models has confirmed the ability to discriminate between immediate and delayed effects [9]–[14], as well as many other aspects of the radiation response (reviewed in [15]–[17]).

We measured mRNA levels in liver tissue, as the liver is a readily accessible and well-characterized organ in small laboratory fish [18], [19]. It is also one of the first sites to show age-associated degenerative changes, which we reasoned might be similar to those induced by low dose radiation [20]. Results allow us to define genes whose expression is altered in an intact vertebrate model, nearly four months following radiation exposure, and to identify some of the biological pathways with which these genes are associated.

## Materials and Methods

### Animal Methods

This study was carried out in accordance with the recommendations in the Guide for the Care and Use of Laboratory Animals of the National Institutes of Health. This study received specific approval from the Institutional Animal Care and Use Committee at Georgia Regents University, formerly Georgia Health Sciences University (protocol number BR09-10-259). Exposure to ionizing radiation at the doses used in this study is not known to cause pain or distress.

We established five experimental groups as follows: zebrafish embryos were collected from three wild type breeding pairs, pooled, and allowed to develop for 24 hours. One group of ∼50 embryos was withdrawn to serve as the non-irradiated control (Group A). To investigate the long-term response, two other groups of ∼50 embryos each were withdrawn and irradiated with 0.1 Gy (Group B) or 1.0 Gy (Group C) of ^137^Cs γ-rays (Model 68A irradiator, J. L. Shepherd & Associates, San Fernando, CA). Embryos in all three groups were then allowed to grow and develop at 28°C using standard maintenance protocols [21]. At 16 weeks post-fertilization, six males from each group were randomly selected, anesthetized with MS-222 (ethyl 3-aminobenzoate methanesulfonate salt; Sigma–Aldrich, St. Louis, MO), sacrificed, sex verified, and dissected. Livers were removed and rapidly frozen in TRIzol Reagent (Life Technologies, Grand Island, NY). In addition, 12 males from Group A were randomly selected and used to establish the acute response groups. These were subjected to whole body irradiation at 0.1 Gy (Group D) or 1.0 Gy (Group E) and sacrificed for analysis at 4 hours post-irradiation. Females were excluded because the role of liver in oogenesis makes hormonal variation a potential confounding factor.

### RNA Isolation and Microarray Hybridization

We performed microarray analysis using three replicates from each group (three pools of two fish each). Sample preparation and analysis were performed in the Georgia Regents University Cancer Center Integrated Genomic Core. Total RNA extraction, cDNA synthesis, and synthesis and labeling of antisense RNA was performed as recommended by Affymetrix using Life Technologies kits. Microarray analysis was performed using the GeneChip Zebrafish Genome Array (Affymetrix, Santa Clara, CA). Primary data have been deposited in GEO (Accession Number GSE46026).

### Statistical Methods

The quality of the data for all chips was evaluated using probe-level models, using the affyPLM package in the R statistical computing environment [22], [23]. These data were then normalized using quantile normalization, and probe set expression values were calculated using the Robust Multiarray Average method [24], [25]. Any probe sets having an interquartile range less than 0.2 were filtered out for all subsequent analyses. The Linear Models for Microarray Data (LIMMA) package [26] was used to determine which probe sets differed among the five treatment groups using comparisons enumerated in the Results section. Only probe sets that had a significant result for the overall F-test were considered significant for any of the comparisons to protect against increasing errors due to increasing multiple comparisons. A 5% false discovery rate (FDR) [27] was used to determine significance for any given test.

Pathway-based analyses were conducted using multivariate analysis of variance using the sets of genes belonging to the same Kyoto Encyclopedia of Genes and Genomes (KEGG) pathway. Classical multidimensional scaling was used to represent the variation in all genes within the gene set using only two dimensions and Euclidean distances. Permutations were used to test significance, and a 5% FDR was used to adjust for multiple testing. This is an extension of Hoteling’s T^2^ approach [28], [29].

### Quantitative Real-time PCR (qPCR)

Synthesis of cDNA was performed using a OneStep RT-PCR kit (QIAGEN, Valencia, CA) with 1 µg of RNA as input. qPCR was performed using a QuantiTect SYBR Green RT-PCR kit (QIAGEN). Gene-specific primers are listed in Table S3. The ΔΔC_t_ method was used for calculation and analysis, with the RPL13a gene as the internal reference.

## Results

### Experimental Design and Overview of Results

**Figure 1:**
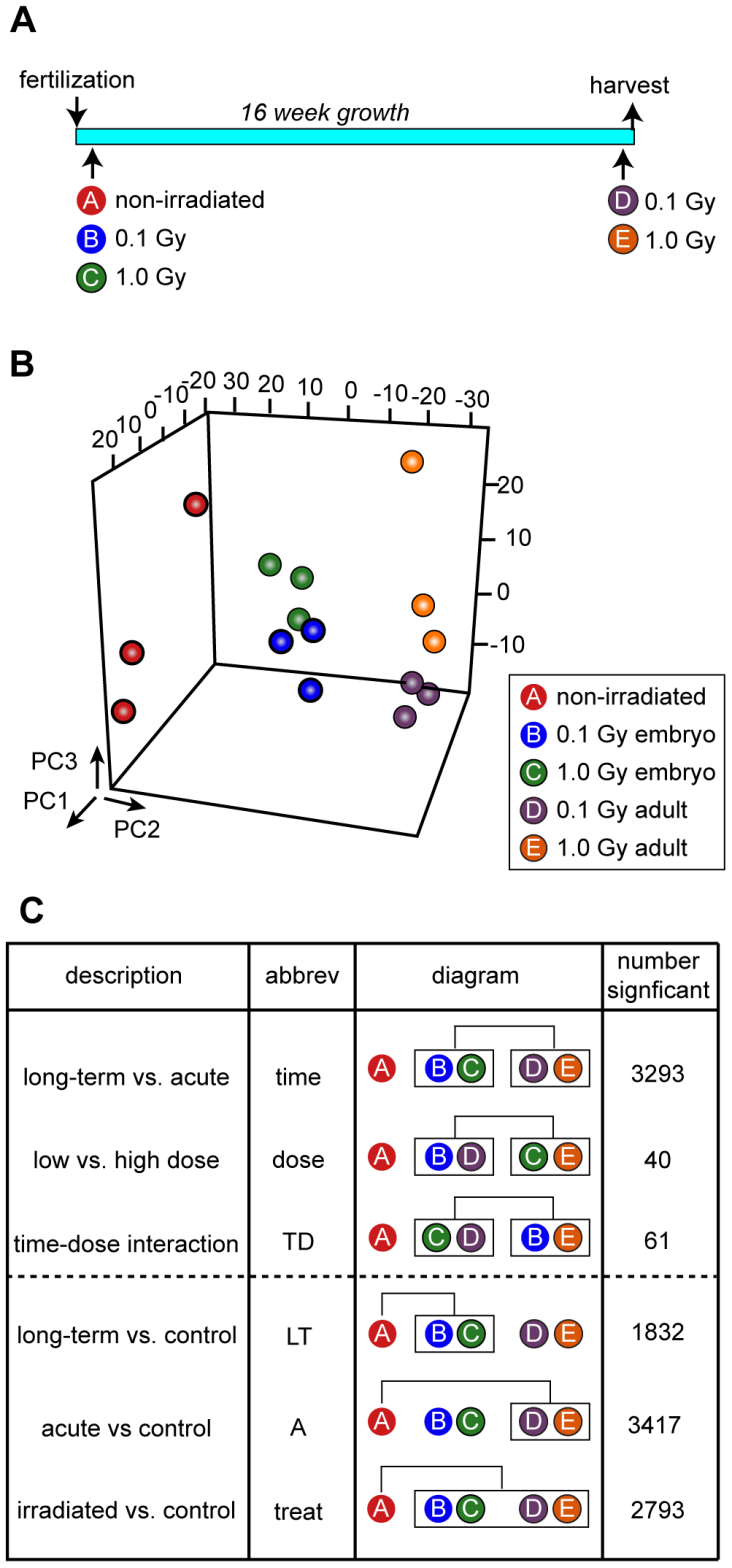
Experimental design and overview of results. A. Design. There are five experimental groups, which were established as detailed in Materials and Methods. Group A, non-irradiated control, Groups B and C, long-term response, Groups D and E, acute response. B. Multidimensional scaling representation. Three-dimensional plot shows three biological replicates per experimental group. PC, Principal Coordinates. Color key as shown. C. Inter-group comparisons. The first three comparisons, which were set up in a way to minimize the effects of multiple testing, evaluate the effect of time of irradiation, dose, and time-dose interaction. Three further comparisons identify genes that are significant comparing long-term response samples as a group versus control samples, acute response samples as a group versus control samples, and all irradiated samples as a group versus control. The number of probe sets identified as significant in each comparison is indicated.

The goal of this work was to define the long-term response to low and moderate dose radiation exposure and to contrast it with an acute response in the same tissue. To do this, we chose a design where the age at analysis was fixed and the age at exposure varied. For convenience, we shall refer to individuals exposed as embryos and analyzed after 16 weeks as the “long-term response groups” and those that were exposed as 16 week old adults and analyzed after four hours as the “acute response groups,” although in actuality, the treatment groups differ both with regard to the duration of the response and the age at exposure, these variables being linked. The strategy used to establish the treatment groups is diagrammed in Figure 1A and is explained in detail in Materials and Methods. Upon completion of the treatments, we harvested liver tissue, extracted RNA, and performed microarray analysis using three biological replicates (pools of two fish each) from each treatment group.

An overview of the differences between individuals, reduced to three dimensions based on multidimensional scaling, is plotted in Figure 1B. The first dimension mainly separates the irradiated from non-irradiated groups, the second dimension mainly separates based on timing (i.e., long-term versus acute response groups), and the third dimension mainly separates based on dose. These results suggest that the largest difference between irradiated and non-irradiated groups relates to timing, with the dose effect being smaller. In particular, the individuals in the two long-term response groups clustered close to one another, regardless of whether they received 0.1 or 1.0 Gy.

We next performed comparisons at the individual gene level (Figure 1C). To investigate the different responses that occurred with different radiation treatments, we set up three comparisons in a way to minimize the effect of multiple comparisons. One was a time comparison, to identify genes where expression in the long-term groups differed from the acute groups; another was a dose comparison, to identify genes where expression in the 0.1 Gy groups differed from the 1.0 Gy groups; and a third was a time-dose interaction comparison, to identify genes where the difference between long-term and short-term exposure groups depended on the radiation dose. Results indicated that many probe sets showed a time effect (3293 of 5307 probe sets with detectable hybridization), whereas fewer probe sets showed a dose effect (40 of 5307) or a time dose interaction (61 of 5307).

To further narrow the scope of investigation, we set up three additional comparisons between treated groups and non-irradiated controls (the first set of comparisons did not involve the control group). One of these was between the long-term response groups and the control group, another was between the acute response groups and the control group, and a third was between the irradiated groups, taken together, and the control group (Figure 1C). Results from this second set of comparisons were used as a filter to identify which changes in gene expression, among those that were identified as statistically significant in the first set of comparisons, were also biologically significant.

### Different Transcripts Associated with Long-term and Acute Responses

**Figure 2:**
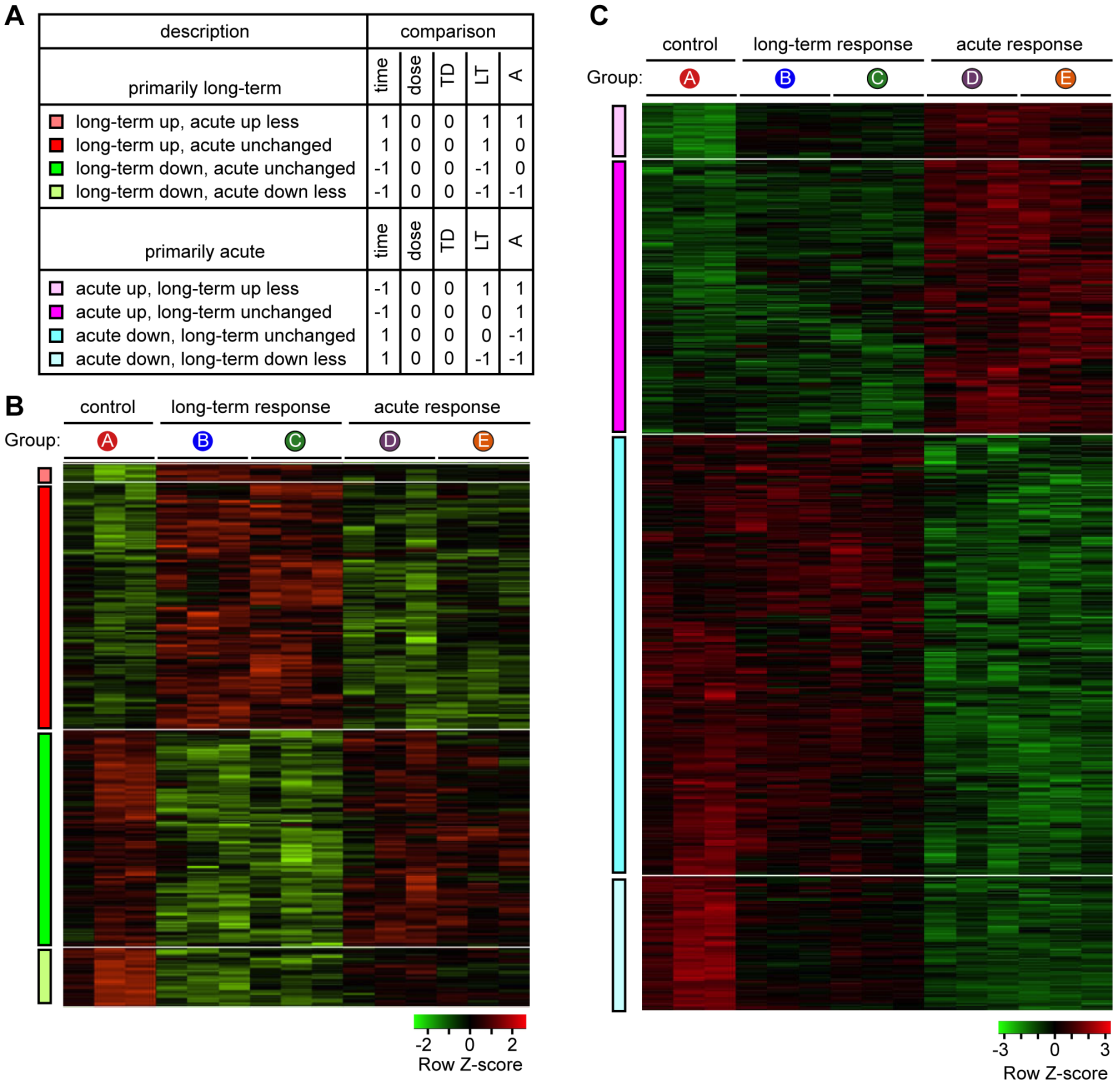
Probe sets associated with long-term and acute responses. A. Inclusion criteria used to define probe sets as primarily or exclusively associated with long-term or acute radiation responses. Statistical comparisons and their abbreviations are as diagrammed in Fig. 1. A “1” indicates that expression of the first set in the comparison was greater than the second; a “−1” indicates that expression in the second set was greater, and a “0” indicates that the difference in expression was not significant. B. Heat map of probe sets primarily associated with long-term response. Red, higher expression; green, lower expression. Each column represents a biological replicate, and each row represents a probe set. C. Heat map of probe sets primarily associated with acute response.

We developed criteria for assigning probe sets as associated primarily or exclusively with the long-term or acute response (Figure 2A, see figure legend for explanation). The inclusion criteria were that expression differed in the time comparison *and* in at least one other comparison: long-term response versus control *and/or* acute response versus control. For clarity, we excluded a small number of probe sets with outlying or rare expression patterns, including those with significant dose dependence, time-dose interaction, or discrepant acute and long-term responses. Because there were so few probe sets with these patterns, their exclusion in the initial analysis does not affect the overall conclusions.

Based on these inclusion criteria, we created the heat maps in Figures 2B and 2C, depicting expression levels of transcripts that were associated primarily with the long-term response or acute responses, respectively. Affymetrix probe identifiers, gene symbols, gene ontologies, fold change, and adjusted *P* values for each of the genes in the figure are provided in Table S1 (long-term response) and Table S2 (acute response). It is evident from the heat maps that the long-term and acute responses are quite distinct, in the sense that many genes can be assigned primarily to one or the other, not both. This suggests that the biological mechanisms underlying the long-term and acute response transcriptional responses are different.

### Genes Highly Affected by Long-term and Acute Responses

The 10 most up-regulated and 10 most down-regulated transcripts associated with the long-term response are shown in Table 1. We omitted duplicate probe sets and genes of unknown function from this table, although these may be found in the more comprehensive Table S2. Many of the up-regulated transcripts are associated with cell signaling or gene regulation. Two are SH2-containing suppressors of cytokine signaling (cish and socs8). Two others are associated with the NF-κB pathway (nfkbiaa and nfkb2), which is an important contributor to non-targeted and inflammatory responses to radiation in mammals [30], [31]. Other up-regulated transcripts include a nuclear receptor (nr1d2b), a G protein alpha subunit (gnai2), and a growth factor (igf2a). Two of the down-regulated transcripts are proline hydroxylases, involved in regulation of hypoxia-induced factor activity (egln3) and collagen biosynthesis (p4ha1) respectively. Most of the others are involved in aspects of energy metabolism or protein biosynthesis.

**Table 1:** Genes increased or decreased as part of the long-term response to radiation.

Rank	Symbol	Gene name	Fold increase	*P* value	Acute	Function
1	cish	cytokine inducible SH2-containing protein	5.08	0.0228	unchanged	cytokine signaling
2	socs8	suppressor of cytokine signaling 8	3.15	0.0454	unchanged	cytokine signaling
3	mylk3	myosin light chain kinase 3	2.51	0.0003	up, less	heart morphogenesis
4	nfkbiaa	NFκB inhibitor, alpha a	2.26	0.0026	up, less	regulation of transcription
5	nr1d2b	nuclear receptor subfamily 1, group D, member 2b	2.15	0.0004	up, less	regulation of transcription
6	col5a1	procollagen, type V, alpha 1	2.10	0.0009	unchanged	cell adhesion
7	gnai2	G protein alpha inhibiting activity polypeptide 2	1.92	0.0072	unchanged	signal transduction
8	serac1	serine active site containing 1	1.79	0.0009	unchanged	GPI anchor
9	nfkb2	NFκB, p49/p100	1.77	0.0037	unchanged	regulation of transcription
10	igf2a	insulin-like growth factor 2a	1.70	0.0259	unchanged	somitogenesis (IGF receptor binding)
**Rank**	**Symbol**	**Gene name**	**Fold decrease**	***P*** ** value**	**Acute**	**Function**
1	egln3	egl nine homolog 3	6.90	0.0134	unchanged	proline hydroxylation
2	p4ha1	Proline-4-hydroxylase	4.72	0.0130	unchanged	proline hydroxylation
3	hsd17b12a	hydroxysteroid (17-beta) dehydrogenase 12a	3.59	0.0005	down, less	steroid biosynthesis
4	sult1st1	cytosolic sulfotransferase 1	3.57	0.0064	unchanged	catecholamine metabolism
5	atp1b2a	ATPase, Na+/K+ transporting, beta 2a polypeptide	2.99	0.0006	down, less	transporter
6	alg2	asparagine-linked glycosylation 2 homolog	2.51	0.0015	unchanged	glycosylation
7	pgp	phosphoglycolate phosphatase	2.33	0.0416	unchanged	energy metabolism
8	eif42a	eukaryotic translation initiation factor 4, gamma 2a	2.31	0.0026	unchanged	protein biosynthesis
9	dlat	pyruvate dehydrogenase (E2 component)	2.26	0.0006	down, less	energy metabolism
10	ippk	inositol 1,3,4,5,6-pentakisphosphate 2-kinase	2.24	0.0006	down, less	organismal development

Top 10 genes that were increased and top 10 genes that were decreased in association with the long-term response to radiation, drawn from Table S1. Gene symbols, gene names, fold change, and *P* values (in the “time comparison”, Fig. 1C) are given. Expression of the genes listed here was either unchanged, or changed less, in the acute response (as indicated in column labeled “Acute”). Some gene names and functions have been edited for clarity or brevity. Genes without a substantive common name, for which biological process is not annotated, have been omitted. Genes that are represented by more than probe set are listed only once.

A similar list of genes most affected in the acute response is presented in Table 2. The well-studied endoplasmic reticulum stress factor, hspa5 (also known as GRP78) was strongly down-regulated. A zebrafish gene related to the hypoxia-induced factor (HIF)-3α was strongly up-regulated, and the liver-specific tumor suppressor, C/EBP alpha [32] was strongly down-regulated. This behavior has a parallel in mammals, where there is a hypoxia-induced transcription factor-C/EBP “signaling axis” characterized by reciprocal regulation of these genes [33].

**Table 2:** Genes increased or decreased as part of the acute response to radiation.

Rank	Symbol	Gene name	Fold increase	*P* value	Long-term	Function
1	c7	complement component 7	23.58	<0.0001	unchanged	complement
2	LOC100330542	Hif3a-like	21.18	<0.0001	up, less	regulation of transcription
3	igfbp1b	insulin-like growth factor binding protein 1b	15.22	<0.0001	unchanged	regulation of cell growth
4	cpt1b	carnitine palmitoyltransferase 1B (muscle)	12.70	<0.0001	unchanged	lipid metabolism
5	glula	glutamine synthase a	10.12	<0.0001	up, less	glutamine biosynthesis
6	crb2	crumbs homolog 2	9.42	0.0003	unchanged	brain development
7	mknk2b	MAP kinase-interacting serine/threonine kinase 2b	9.41	<0.0001	up, less	regulation of translation
8	thraa	thyroid hormone receptor alpha a	8.69	<0.0001	up, less	regulation of transcription
9	ucp2	uncoupling protein 2	7.91	<0.0001	unchanged	transport
10	histh1	Histone H1 like	7.42	<0.0001	up, less	chromatin component
**Rank**	**Symbol**	**Gene name**	**Fold decrease**	***P*** ** value**	**Long-term**	**Function**
1	hspa5	heat shock protein 5	29.19	<0.0001	down, less	response to stress
2	cebpa	C/EBP alpha	13.19	<0.0001	down, less	regulation of transcription
3	dynll1	dynein, light chain, LC8-type 1	7.31	<0.0001	down, less	microtubule-based process
4	pdia4	protein disulfide isomerase associated 4	6.21	<0.0001	down, less	glycerol ether metabolism
5	cask	calcium/calmodulin-dependent serine protein kinase	5.86	<0.0001	down, less	protein phosphorylation
6	calrl	calreticulin like	5.34	<0.0001	down, less	protein folding
7	prox1/prox2	prospero-related homeobox gene 1/2	5.22	<0.0001	down, less	lymphangiogenesis
8	ghrb	growth hormone receptor b	4.91	<0.0001	unchanged	hormone receptor
9	cpox	coproporphyrinogen oxidase	4.69	<0.0001	down, less	porphyrin biosynthesis
10	mlec	malectin	4.67	<0.0001	down, less	protein N-glycosylation

Top 10 genes that were increased and top 10 genes that were decreased in association with the acute response to radiation, drawn from Table S2. Gene symbols, gene names, fold change, and *P* values (in the “time comparison”, Fig. 1C) are given. Expression of the genes listed here was either unchanged, or changed less, in the long-term response (as indicated in column labeled “Long-term”). Some gene names and functions have been edited, and some have been omitted, as in Table 1.

The dynamic range of the transcriptional effect was greater in the acute than in the long-term response groups. Thus, in the acute response groups, the most highly affected genes changed by ∼25 to 30-fold, whereas in the long-term response groups, the most highly affected genes changed by ∼5 to 7-fold. Perhaps, this reflects a tendency of the tissue to return to homeostasis over a 16 week post-irradiation recovery period, as compared to a 4 hour recovery period.

### Validation by Quantitative PCR

To confirm the validity of the microarray data by an independent method, we measured expression of five transcripts by quantitative PCR, including three that were associated with the long-term response, cish, socs8, and nfkb2, and two that were associated exclusively or primarily with acute response, igfbp1b and hspa5. The transcripts that were selected for validation met two criteria: they showed a large fold change, relative to control, for at least one irradiated group, and they had perceived biological relevance to the radiation response. Table S3 gives the primer sequences that were used.

**Figure 3:**
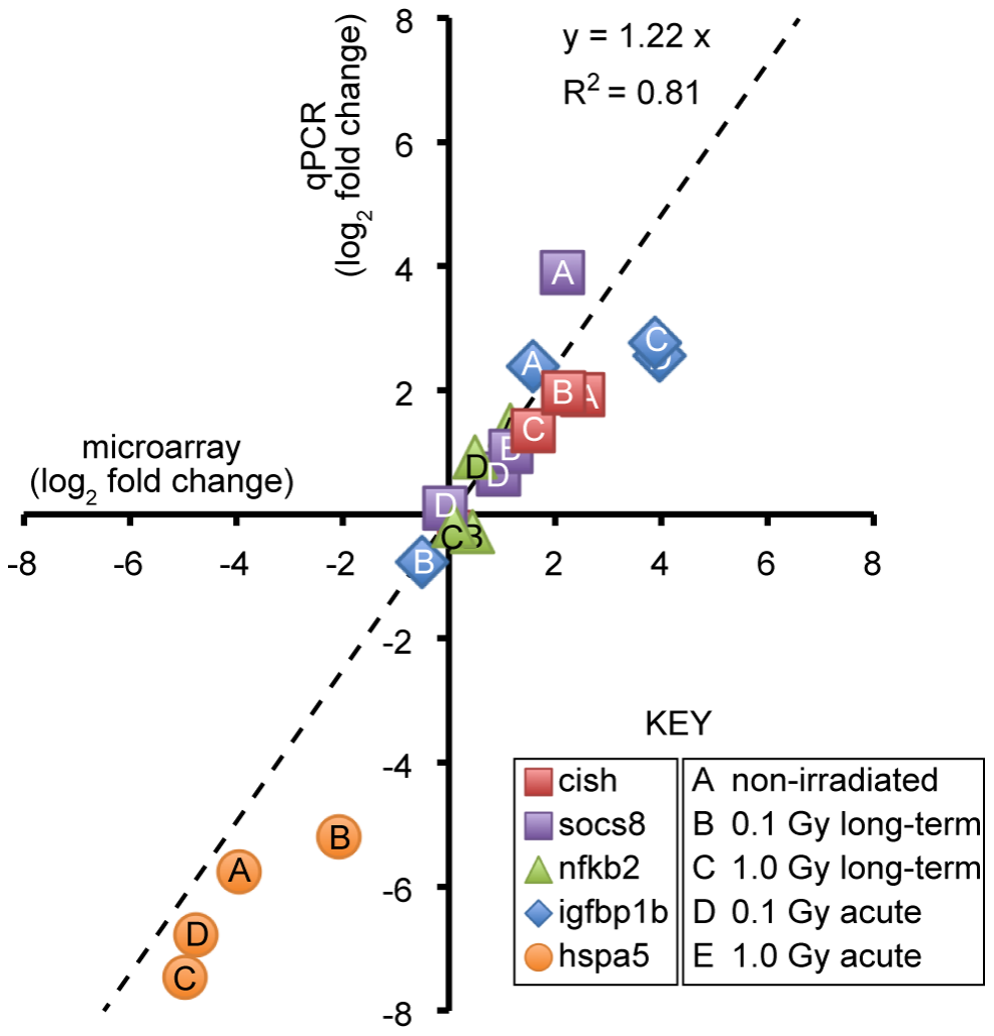
Validation of microarray data by quantitative polymerase chain reaction. Scatter plot shows fold change on logarithmic scale as measured for selected genes by microarray and qPCR. Each point represents mean value for one gene and experimental group. Shape and color denotes gene; letter indicates experimental group as indicated.

A scatter plot (Figure 3) shows good agreement between mean expression levels as measured by gene expression profiling and by qPCR, with a coefficient of determination, R^2^, of 0.81. The changes in mRNA levels as measured by qPCR were consistently about 1.2-fold greater than as measured by microarray (note slope of trend line). If one assumes that qPCR is the more accurate method, then the microarray data slightly underestimate the actual effects of treatment.

### Genes in the p53 Signaling Pathway are More Affected in the Acute than in the Long-term Response

To gain further insight into the biological significance of the changes in gene expression, we sought to identify KEGG pathways that were significantly affected by radiation treatment. Given the widespread effects of treatment on gene expression, and the fact that many KEGG pathways contain overlapping gene sets, it is perhaps unsurprising that significant changes were seen in many pathways –122 in all. Most commonly, pathways were affected in both the long-term and acute responses, although the patterns of change differed and many individual genes within each pathway could be assigned primarily to one response or the other. We selected two pathways to discuss in detail, the KEGG p53 signaling pathway (http://www.genome.jp/kegg-bin/show_pathway?dre04115) and the KEGG apoptosis pathway (http://www.genome.jp/kegg-bin/show_pathway?dre04210). These were chosen based on the fraction of genes affected within each pathway and the evident relevance of these two pathways to radiation biology.

**Figure 4:**
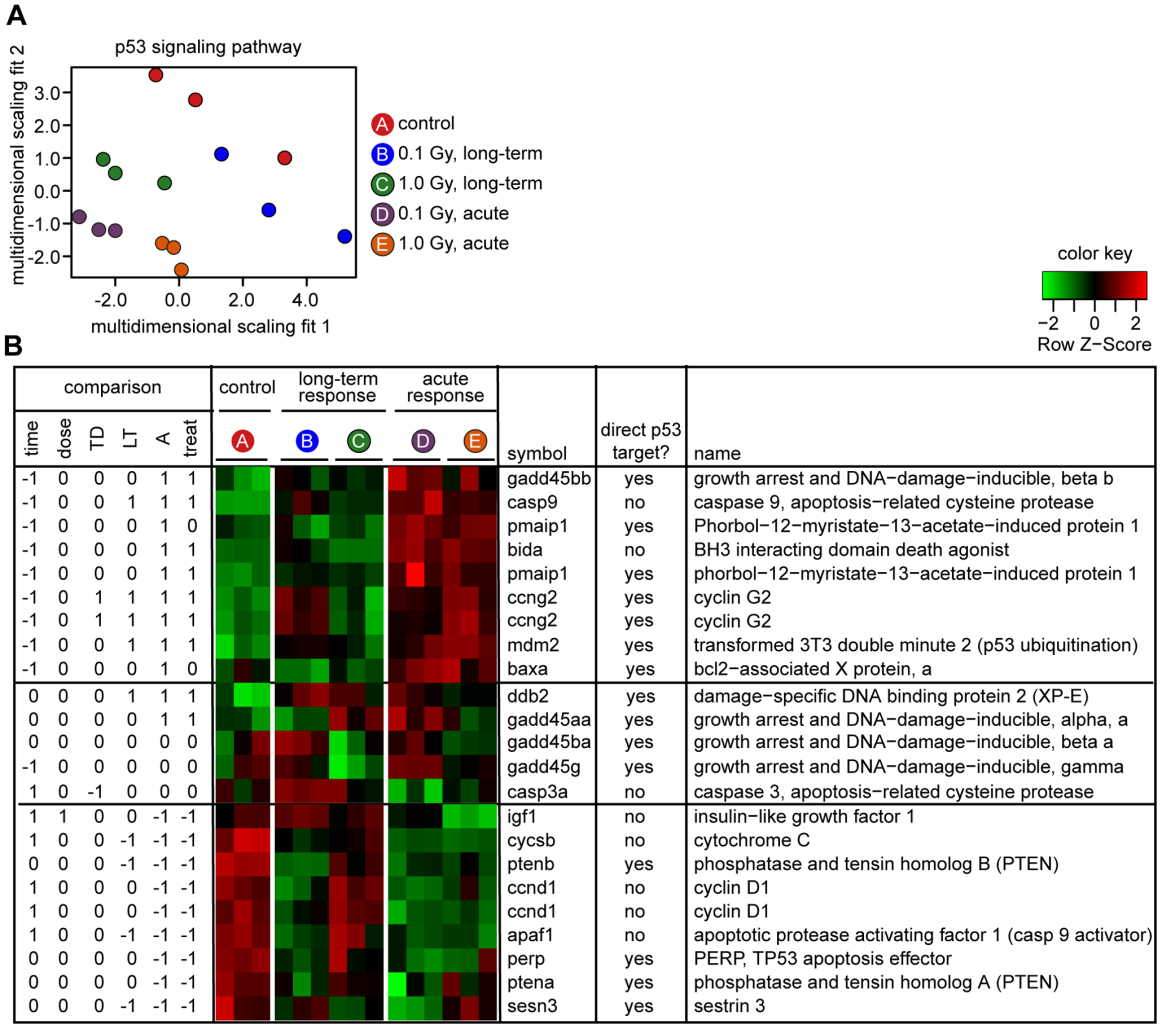
Analysis of p53 signaling pathway. A. Multidimensional scaling representation of results for the p53-signaling pathway, based on 36 probe sets for which data are available. Each symbol represents one biological replicate. Color denotes treatment group as indicated. B. Response for 23 probe sets that showed statistically significant differences in one or more comparisons. (Results of comparisons are expressed as “1”, “0” or “−1” using the same convention as in Figure 2). One gene (gadd45ba) was included because it was significant in an overall F test, although not in any of the individual comparisons. Left, results of statistical comparisons, notation as in Figures 1 and 2. Center, heat map, as in Figure 2. When more than one row has the same gene symbol, it indicates different probe sets directed against the same mRNA. Right, gene symbols, whether gene is a direct target of the p53 transcription factor, and gene name. In some cases, an alternative name or description of gene function is given in parentheses. For details of KEGG p53 signaling pathway – Danio rerio (zebrafish): http://www.genome.jp/kegg-bin/show_pathway?dre04115.

The KEGG p53 signaling pathway controls DNA-damage dependent cell cycle arrest and apoptosis. Expression was detected for transcripts corresponding to 36 probe sets. Changes were highly significant (*P* = 0.0013 for the acute response, *P* = 0.0060 for the long-term response). An overview of the differences between individuals, reduced to two dimensions based on multidimensional scaling, is plotted in Figure 4A. Individuals in the acute response groups clustered together and were the most distant from the control individuals in this representation. Individuals in the long-term response groups also differed from control, although less markedly.

There were 23 probe sets that, individually, showed significant differences in at least one statistical comparison. Figure 4B shows results of statistical comparisons, a heat map depicting the patterns of expression, and other information (refer to Table S4, for further details about the probe sets, the corresponding genes, and the statistical analysis). We divided the heat map into three sections, based on common patterns of gene expression. Transcripts corresponding to the top nine probe sets were highly induced in the acute response, and were affected more modestly, if at all, in the long-term response. Seven represent direct p53 transcriptional targets as defined in the KEGG pathway map; the other two are presumed to be indirect targets. Genes induced as part of the acute response included many well-known participants in the DNA damage response, including a member of the growth arrest and DNA damage-inducible gene family (gadd45bb), the pro-apoptotic baxa and bida genes, and the mdm2 E3 ubiquitin ligase, which participates in an autoregulatory loop that controls p53 protein levels.

Transcripts corresponding to a middle group of five probe sets showed intermediate or mixed patterns of expression, including two with a general treatment effect and one with time-dose interaction. One gene (gadd45ba) was not significant in any of the individual comparisons but was significant in an overall F test, and for this reason is included.

Transcripts corresponding to the bottom group of nine probe sets were significantly down-regulated in the acute response. In four instances, the results were unexpected, as the mammalian homologs of these genes are direct targets of p53 transcriptional induction (ptena, ptenb, sesn, and perp). It could be that these are *bona fide* p53 targets in the zebrafish, but the induction occurs before or after the single four-hour time point that was analyzed. Alternatively, the down-regulation of these genes could reflect species- or tissue-specific differences in transcriptional control.

Interestingly, among the genes listed as part of the KEGG p53 signaling pathway in zebrafish, none were primarily or exclusively associated with the long-term response. By contrast, there were many genes that were primarily or exclusively associated with the acute response (using the same inclusion criteria as in Figure 2). This implies that p53-mediated gene regulation is less of a factor in the long-term than in the acute response, a mechanistic difference that could be an important contributor to the observed differences in the overall pattern of gene expression.

### The Apoptosis Pathway is affected in both the Long-term and Acute Responses

**Figure 5:**
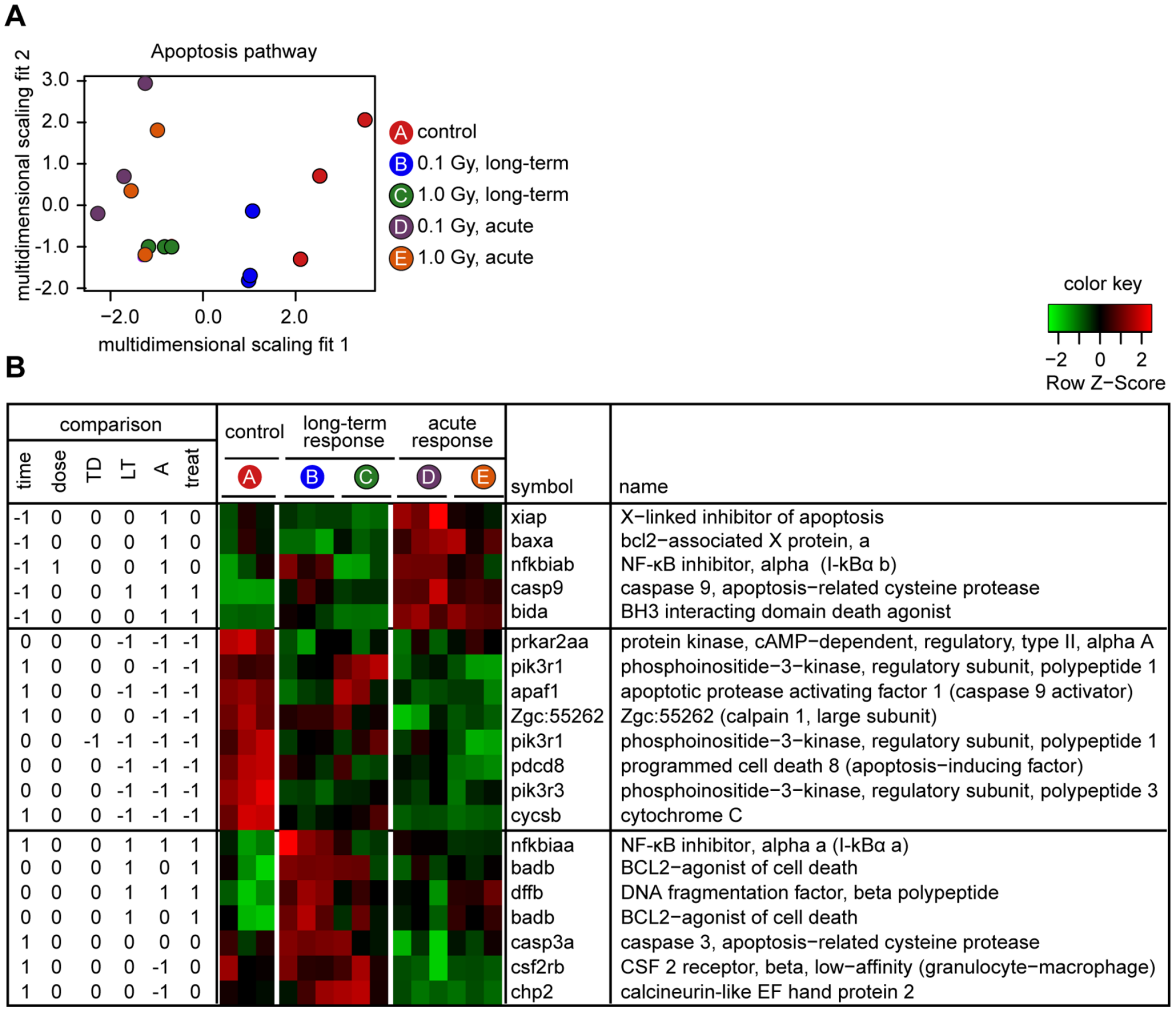
Analysis of apoptosis pathway. A. Multidimensional scaling representation of results for apoptosis pathway, based on 42 probe sets for which data are available, symbols as in Fig. 4. B. Responses for 20 probe sets that showed significant differences in one or more statistical comparisons, depicted and labeled as in Fig. 4. For details of KEGG apoptosis pathway – Danio rerio (zebrafish): (http://www.genome.jp/kegg-bin/show_pathway?dre04210). Note that the name of badb has been corrected to “BCL2-agonist of cell death” (not “antagonist”) in agreement with direct experimental evidence in the zebrafish [34] and the known function of the mammalian ortholog [35].

The KEGG apoptosis pathway includes genes that are important for both the intrinsic or extrinsic mechanisms of apoptosis. Expression was detected for transcripts corresponding to 42 probe sets. A few of these are also included in the KEGG p53 regulatory pathway. The pattern of expression differed between the acute response groups and the control group (*P* = 0.0013), between the long-term response groups and the control groups (*P* = 0.0060), and, unusually, between the high and low dose groups (*P* = 0.048). An overview of the differences between treatment groups, reduced to two dimensions based on multidimensional scaling, is shown in Figure 5A. As with the p53 pathway, individuals in the acute response groups were the most distant from the control individuals in this representation, but in contrast to the p53 pathway, there was some overlap between groups; notably, individuals in long-term 1.0 Gy group and the acute 1.0 Gy group were intermingled.

There were 20 probe sets that, individually, showed significant differences in at least one statistical comparison. Figure 5B shows results of statistical comparisons, a heat map depicting the patterns of expression, and other information (refer to Table S5 for further details). We again divided the heat map into three sections vertically. Transcripts corresponding to the top five probe sets were highly induced in the acute reponse. Among these are the pro-apoptotic genes baxa, bida, and casp9. Potentially offsetting the effects of these genes, there was also strong induction of the anti-apoptotic gene, xiap. Another gene in this group, interestingly, is nfkbiab, which encodes a different isoform of the IκBα factor than the one previously discussed in the context of the long-term response.

Transcripts corresponding to a middle group of eight probe sets were down-regulated in the acute response and, in most cases, in the long-term response. These genes include cytochrome c, a factor involved in cytochrome c release (pdcd8), and a number of upstream apoptotic regulatory genes.

Transcripts corresponding to the bottom group of seven probe sets showed a high level of expression in the long-term response groups relative to control (three probe sets), relative to acute groups (three probe sets) or relative to both (one probe set). This bottom group includes the pro-apoptotic bad gene, a common subunit of several cytokine receptors (csf2rb), an enzyme responsible for apoptotic DNA fragmentation (dffb), a phosphatase regulatory protein (chp2) and, as discussed previously, an isoform of IκBα that differs from the one induced in the acute response (nfkbiaa).

## Discussion

We describe here the use of the zebrafish model to investigate the long-term effects of radiation exposure, based on changes in the mRNA expression pattern in the liver. The study was performed using the Affymetrix microarray platform, with technical validation of the results for selected transcripts using qPCR. Distinctive aspects of the study were that it characterized the radiation response *in vivo* in an intact organism, that it characterized low-dose effects, and that it characterized long-term effects, that is, where there was a long time interval between exposure and analysis. The biological characteristics of the zebrafish model, where development occurs external to the mother, were an enabling factor for this particular design, where embryos but not mothers were exposed.

The study reached three main conclusions (1) there is a large set of transcripts where a low-dose exposure early in life affects expression in the adult, many months following the initial treatment, (2) this long-term response is not merely an attenuated version of an acute response, but involves hundreds of genes that are unaffected or less affected when individuals of the same age were irradiated 4 hours before analysis, and (3) the effects of 0.1 Gy and 1.0 Gy exposure are in many instances similar. It is important to qualify the last of these conclusions by noting that zebrafish, with their compact genome, are more radioresistant than mammals. Radiation cytotoxicity to the embryo at these doses is either minimal (0.1 Gy) or mild and well tolerated (1.0 Gy). Thus, both doses might be within the “low-dose” regime in the zebrafish model.

The marked difference between long-term and acute responses points to differences in the underlying mechanisms of transcriptional regulation. It appears that one of these involves p53, which appears to play a much larger role in the acute than the long-term response. The p53 protein is regulated post-translationally (tp53 mRNA was unaffected by treatment in the zebrafish model, data not shown). We did not attempt to measure levels or modifications of p53 directly, as we lacked the necessary species cross-reactive antibodies. Nevertheless, up-regulation of a large number of p53 target genes strongly implies up-regulation of p53 protein itself.

Whereas the p53-dependent DNA damage response appears to contribute to the acute effects of radiation on the mRNA profile, we were not able to identify any single mechanism as a driver of the long-term response. There was a preponderance of signaling and regulatory genes among the up-regulated transcripts and of metabolic enzymes among the down-regulated transcripts. One hypothesis is that radiation exposure sets in motion long-term inflammatory processes, to the detriment of liver parenchymal cell function. However, the specifics are not well understood, in part because not all of the genes that regulate inflammatory responses are well characterized in the zebrafish. An example is socs8, which was among the most highly induced transcripts in the long-term response. In mammals, members of the socs gene family inhibit stat proteins. However, there is apparently no precise mammalian ortholog of socs8, and its exact role in stat regulation remains to be elucidated.

Apoptotic regulatory genes were affected in the long-term response, although the pattern of change was different than in the acute response. Of note, the bad gene was highly induced in the long-term response. In zebrafish and other organisms, bad is pro-apoptotic [34]. In mammals, its product both competes with anti-apoptotic bcl-2 family members and forms a complex with p53 protein that permeabilizes the mitochondrial membrane [35]. In addition to up-regulation of bad, we found that chp2 was expressed at higher levels in the long-term response groups than in the acute response groups. As indicated in the KEGG apoptosis pathway chart, chp2 influences protein phosphatase 3 activity, which promotes removal of a phosphate group that negatively regulates activity of the bad product. Thus, both transcriptional and post-translational mechanisms may play into the activity of bad in the long-term response.

One of the interesting features of zebrafish, and of teleost models in general, is an ancient, whole-genome duplication that created many homeologous genes, some of which have evolved functional differences and others not. For example, induction of nfkbiab was associated with the acute response, whereas induction of nfkbiaa was associated primarily (but not exclusively) with the long-term response. Similarly, induction of gadd45bb was associated with the acute response, whereas induction of gadd45ba showed a mixed pattern with a trend toward inverse dose-dependence. It will be of interest to learn whether these pairs of homeologous genes have evolved different functions, in addition to different regulatory patterns.

One of the features that make zebrafish attractive as a model organism is the ability to manipulate embryonic gene expression via microinjection with mRNA or antisense morpholino oligonucleotide. As an example, knockdown of Ku70 or Ku80 repair proteins sensitizes embryos to acute radiation-induced apoptosis, an effect that can be reversed by co-injection of morpholino oligonucleotide-resistant mRNA [2], [36]. Although we did not take advantage of this feature of the model in the present study, it should be readily possible to do so in the future, for example, by attenuating expression of inflammatory signaling genes to determine if this influences the long-term response as reflected in the pattern of organ-specific gene expression.

### Conclusions

More than 350 transcripts are distinctively altered in the livers of adult zebrafish liver, as measured 16 weeks following low or moderated dose radiation treatment of zebrafish embryos. The long-term response differs strikingly from that seen when adults of the same age were irradiated four hours before analysis. Results indicate that the zebrafish holds promise as a genetically tractable model for addressing mechanisms by which radiation injury translates into long-term effects.

## Supporting Information

Table S1 Figure 2B gene list. List of genes that appear in the heat map in Figure 2B. Columns contain: Affymetrix probe set ID, gene symbol, gene title, Entrez gene number, Gene Ontology (Biological Process, Cellular Component, or Molecular Function), gene group in this experiment (i.e., whether the probe set was significantly up or down comparing the long-term response groups with the non-irradiated control group, and whether or not it also showed a change in the acute response group relative to the control group), the fold change up or down for the comparison between the long-term response groups and the control group (note that fold change up and fold change down are reciprocals of one another), and the adjusted *P* value for the comparison of the long-term response group and control group.
(XLSX)

Table S2 Figure 2C gene list. List of genes that appear in the heat map in Figure 2C. Column labels are the same as for Table S1, except that comparisons are for the acute groups versus the control group.
(XLSX)

Table S3 Primers for qPCR. List of primers used for experiment in Figure 3. Primers were used to amplify cDNA for indicated genes.
(XLSX)

Table S4 Figure 4 gene list. List of genes that appear in the heat map in Figure 4. Columns A-G contain: Affymetrix probe set ID, gene symbol, gene title, Entrez gene number, Gene Ontology (Biological Process, Cellular Component, or Molecular Function). The remaining columns contain results for the six statistical comparisons diagrammed in Figure 1: treatment, time, dose, time-dose interaction, long-term versus control, and acute versus control. For each of the comparisons, the log-transformed difference, the fold change up or down, and the adjusted *P* value are given.
(XLSX)

Table S5 Figure 5 gene list. List of genes that appear in the heat map in Figure 5. Column labels are the same as for Table S4. Note that gene name for badb has been corrected to “BCL2-agonist of cell death” as explained in the legend for Figure 5 in the main text.
(XLSX)
