# Correction: Long-Term Effects of Ionizing Radiation on Gene Expression in a Zebrafish Model

**DOI:** 10.1371/annotation/4a4ddb44-b142-4001-bab0-8012da225d65

**Published:** 2014-01-21

**Authors:** Lahcen Jaafar, Robert H. Podolsky, William S. Dynan

The qPCR validation experiment in Figure 3 used primers directed against the igfbp1a gene, not igfbp1b. The qPCR values should thus have been plotted against igfbp1a microarray data. 

In Figure 3, the correct slope of the regression line is 1.25. The correct coefficient of determination (r^2^) is 0.89. The points labeled A,B,C, and D are also incorrect. The correct version of Figure 3 can be viewed here: 

**Figure:**
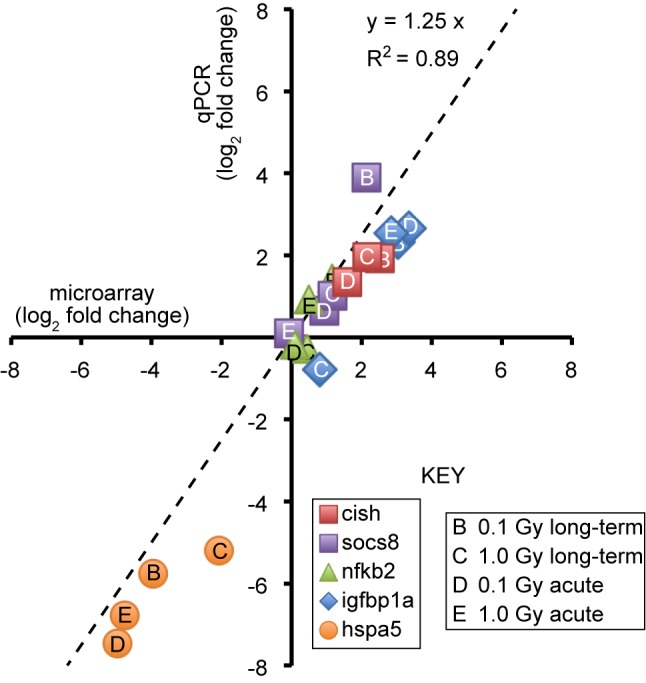


In addition, in Table S3, primers labeled “igfbp1b” should be labeled “igfbp1a" and the sequence of the forward primer is d(AGTCAATGAAGGCAGCTCC), one nucleotide longer than originally given.

